# Impact of Polymer-Assisted Epitaxial Graphene Growth on Various Types of SiC Substrates

**DOI:** 10.1021/acsaelm.2c00989

**Published:** 2022-11-01

**Authors:** Atasi Chatterjee, Mattias Kruskopf, Stefan Wundrack, Peter Hinze, Klaus Pierz, Rainer Stosch, Hansjoerg Scherer

**Affiliations:** Physikalisch-Technische Bundesanstalt, Bundesallee 100, 38116 Braunschweig, Germany

**Keywords:** epitaxial graphene, polymer-assisted growth, spin-on deposition, miscut angle, SiC, concentration dependent

## Abstract

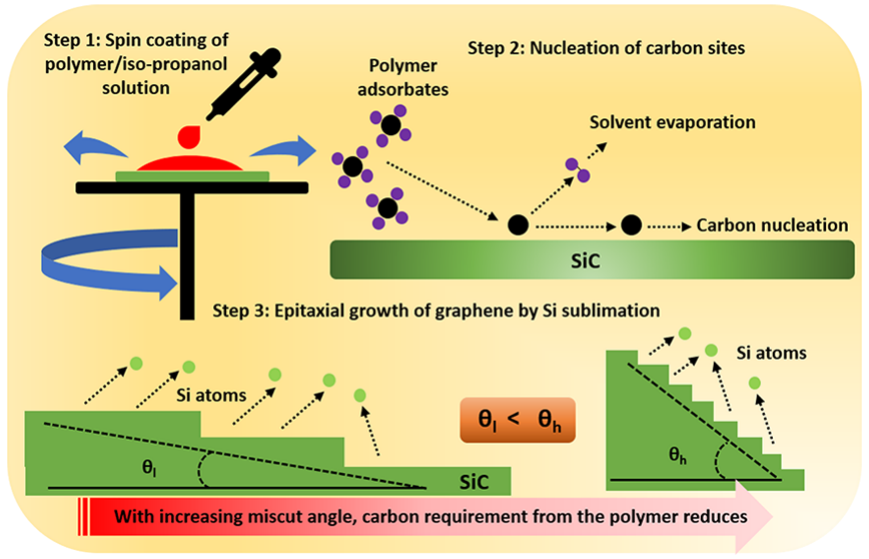

The growth parameters for epitaxial growth of graphene on silicon carbide (SiC) have been the focus of research over the past few years. However, besides the standard growth parameters, the influence of the substrate pretreatment and properties of the underlying SiC wafer are critical parameters for optimizing the quality of monolayer graphene on SiC. In this systematic study, we show how the surface properties and the pretreatment determine the quality of monolayer graphene using polymer-assisted sublimation growth (PASG) on SiC. Using the spin-on deposition technique of PASG, several polymer concentrations have been investigated to understand the influence of the polymer content on the final monolayer coverage using wafers of different miscut angles and different polytypes. Confocal laser scanning microscopy (CLSM), atomic force microscopy (AFM), Raman spectroscopy, and scanning electron microscopy (SEM) were used to characterize these films. The results show that, even for SiC substrates with high miscut angles, high-quality graphene is obtained when an appropriate polymer concentration is applied. This is in excellent agreement with the model understanding that an insufficient carbon supply from SiC step edge decomposition can be compensated by additionally providing carbon from a polymer source. The described methods make the PASG spin-on deposition technique more convenient for commercial use.

## 1 Introduction

Single-layer epitaxial graphene is well-known for its extraordinary structural and electronic properties. Graphene on SiC is a potential candidate in a variety of applications such as radio frequency (RF) transistors, integrated circuits (ICs), field-effect transistors (FETs), and sensors.^1^ Apart from the aforementioned applications, focused studies on graphene-based quantum Hall resistance standards have shown that graphene exhibits quantization at magnetic fields below 5 T and temperatures above 4 K. Such relaxed measurement conditions have made graphene an outstanding candidate for AC and DC quantum metrology applications.^2−5^ Additionally, the fabrication of two-dimensional (2D) heterostructures and the intercalation of graphene layers with other materials have opened routes for engineering new electronic material systems.^6,7^ Nevertheless, for all the requirements mentioned before, consistent methods for producing large-area homogeneous graphene layers of high quality are necessary. Epitaxial growth on SiC substrates fulfills all these requirements and, moreover, avoids the transfer to another substrate and is compatible with existing fabrication technologies.^8−11^ The key prerequisite to obtaining high-quality epitaxial monolayer graphene films is to prevent step bunching of the SiC substrate during growth, since high terrace step edges favor bilayer growth along the edges and deteriorate the electronic properties of the graphene. This is challenging since on one hand high process temperatures are necessary for homogeneous growth of the graphene layer which on the other hand promotes SiC step bunching. Therefore, the control of the process parameters (e.g., temperature, pressure, gas flow)^12^ is a severe issue. Moreover, other factors such as wafer properties (e.g., miscut angle, crystal direction)^8,11,13,14^ as well as pretreatment techniques (e.g., substrate cleaning, etching, annealing)^15−21^ prior to high-temperature growth are crucial for the growth result.

Recently, the so-called polymer-assisted sublimation growth (PASG) technique was developed in our lab, which is able to effectively avoid SiC step bunching by a simple improvement of the existing sublimation growth technique.^10,22,23^ A polymer pretreatment is applied on the SiC substrate that supplies extra carbon during the initial growth stages of the buffer layer^10,22,23^ by thermal decomposition. The accelerated buffer layer growth stabilizes the SiC terraces, and sub-nanometer substrate steps are conserved throughout the whole growth process. Using the PASG technique, ultrasmooth monolayer graphene films can be fabricated, showing excellent resistance isotropy,^23^ and metrology-grade quality is demonstrated by their successful application as quantum Hall resistance standards.^24^ This raises the following questions: to what extent does the intrinsic carbon supply from the thermal decomposition of SiC still play a role in graphene growth and how can the external polymer amount be precisely controlled for homogeneous and reproducible graphene growth?

In this study, we investigate in detail the interplay of the two carbon sources for the PASG technique to obtain precise growth control for the fabrication of high-quality graphene layers. On the one hand, the polymer amount was varied, which directly controls the external carbon supply. On the other hand, by using SiC substrates with different miscut angles, the number of terrace steps was varied. Since the thermal decomposition of the SiC preferably takes place at the terrace step edges, the amount of SiC-related carbon is directly related to the substrate’s miscut angle.^16,25^ Moreover, by using 4H- and 6H-SiC substrates the impact of the polytype on the graphene growth is highlighted.

We show that only by controlling the amount of polymer, the graphene growth can be easily optimized and adapted to the miscut angle of the substrate. A detailed investigation of the spin-on technique is presented, which turns out to be a reliable and reproducible polymer deposition technique compared to the liquid phase deposition (LPD) technique. These findings will facilitate implementation into existing production lines and pave the way for improved graphene device applications.

## 2 Materials and Methods

### 2.1 Substrate Pretreatment and Growth

In this study, three different SiC (0001) substrates of hexagonal polytype were used with small miscut angles of 0.1 and 0.03° against the [1–100] direction (perpendicular to the primary flat), because only this guarantees optimal starting conditions with step heights of about one SiC monolayer (∼0.25 nm). The miscut angle was verified by atomic force microscopy (AFM) inspections. For a reliable comparison of the results, a miscut of about 0° was chosen against the [11–20] direction (perpendicular to minor flat), which avoids a roughening of the terrace edges during growth (see Table 1). All wafers were diced into 5 mm × 10 mm individual pieces.

**Table 1:** Different SiC Wafers Used for This Study with Details of Polytype and Miscut Values

polytype	conducting type	miscut toward the primary flat [1–100] (deg)	miscut toward the secondary flat [11–20] (deg)
6H-SiC (0001)	semi-insulating	–0.1	–0.03
6H-SiC (0001)	semi-insulating	–0.03	0.00
4H-SiC (0001)	semi-insulating	+0.04	0.00

The samples were cleaned in acetone and isopropanol (IPA) prior to the polymer treatment. The PASG technique^22^ was applied in two different ways: liquid phase deposition (LPD)^10,23^ and spin-on deposition.^26^ In the case of LPD, the SiC substrates were ultrasonicated in a highly concentrated solution of AZ5214E photoresist in IPA (25% solution) for 15 min, followed by rinsing the substrate to remove the excess polymer using isopropanol from a spray bottle. Solution concentration, sonication time, and the final rinsing time are the three main parameters that can control the size and amount of carbon adsorbates in the LPD technique that finally influence the quality of monolayer graphene.^10^ The solution concentration (25%) and the sonication time (15 min) were kept constant in this study. Two different rinsing times were used: a shorter (6–8 s) time and a longer (10–12 s) time. Details on optimum rinsing times are provided in the Results and Discussion.

The second way to apply the PASG technique is by spin-on deposition of a weak solution of AZ5214E photoresist and isopropanol on SiC substrates prior to growth. The volume ratios of polymer solutions ({AZ(μL)/IPA(ml)}) used were 5.1, 3.4, 2.2, 1.5, and 0.75 and were named C5–C1, respectively (see Table 2)

**Table 2:** Polymer Concentrations Used in This Study

	
concentration	C5	C4	C3	C2	C1
volume ratio ({AZ (μL)/IPA (mL)})	5.1	3.4	2.2	1.5	0.75

The spin-on process involves spin coating of the highly diluted solutions with a high acceleration ramp to 6000 rpm within 1 s and a final speed of 6000 rpm that is held for 30 s. Immediately after this polymer treatment step, the samples were introduced into the inductively heated reactor for graphene growth.^21^ Initially the reactor was evacuated to 1 × 10^–6^ mbar. Ar gas was introduced at 400 °C with a gas flow of 20 sccm and a heating rate of ∼13 K/s. For graphene growth, the samples were annealed at 1750 °C for 6 min at 1 bar Ar pressure. All of the samples presented in this study were subjected to the same growth parameters of temperature, time, and gas flow for the fabrication of high-quality graphene.

### 2.2 Confocal Laser Scanning Microscopy (CLSM)

CLSM is a fast and efficient way to characterize the quality of the samples.^27^ In this work, we used an Olympus LEXT OLS5100 system equipped with the following objectives: ×2.5, ×5, ×10, ×20, ×50, and ×100. Each objective has the option for an additional digital zoom of up to ×8. Hence, it was possible to image large areas such as 2560 μm × 2560 μm to small areas of 16 μm × 16 μm. The 3D laser scanning microscope is equipped with a 405 nm wavelength violet semiconductor laser that scans in the *X*–*Y* direction and a photomultiplier tube that generates images up to 4096 × 4096 pixels. The laser intensity image combines a series of images in the *Z* direction to create a 2D intensity image.

### 2.3 Atomic Force Microscopy (AFM)

AFM was performed using a Witec Alpha 300 RA and Park NX10 instruments in noncontact mode. AFM achieves simultaneous mapping of the topography, feedback, and phase information, which is a measure of the energy dissipation between the probe and sample, thus providing details on the material and height information. The Point Probe Plus Silicon-SPM-Sensor tips (PPP-NCLR-20 type) manufactured by Nanosensors with a resonance frequency of 146–236 kHz were used for the measurements.

### 2.4 Raman Spectroscopy

The Raman measurements were performed using a LabRAM Aramis (Horiba) confocal Raman spectrometer with an excitation wavelength of 532 nm and diffraction grating of 600 grooves per mm. The full width at half-maximum (fwhm) values of the graphene-specific 2D peak were extracted from the individual Raman spectra using a Lorentzian fitting algorithm. The distribution of the fwhm values of the 2D peak of graphene is plotted in a Raman map (area scans) by integrating 7000 individual Raman spectra. Micro-Raman mappings of 20 μm × 20 μm were recorded for all samples. The lateral resolution was about 0.5 μm.

### 2.5 Scanning Electron Microscopy (SEM)

The SEM measurements were performed using a Supra 40 field emission scanning electron microscope (FESEM) from Carl Zeiss GmbH. This SEM is equipped with Zeiss SmartSEM software (version 5.0708) and two types of detectors an in-lens and a secondary electron (SE) detector for capturing images. To resolve the contrasts between buffer layer, monolayer and bilayer graphene, very low acceleration voltages (around 1 keV) were used. A working distance of 3–3.5 mm and an in-lens detector was used for image capturing.

## 3 Results and Discussion

**Figure 1:**
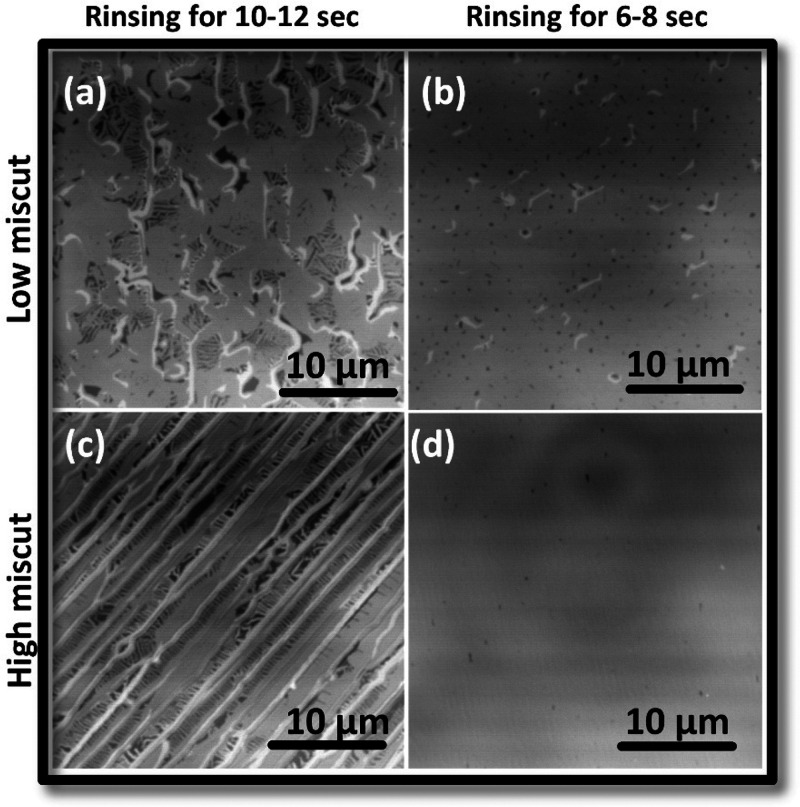
Confocal laser scanning microscopy (CLSM) images of epitaxial graphene samples fabricated by the LPD technique on different miscut angle 6H wafers and two different rinsing times. (a) and (b) Show the growth results of epitaxial graphene on the low-miscut wafer (−0.03°). (c) and (d) show epitaxial graphene grown on the high (−0.1°) miscut wafer. The dark contrast denotes the buffer layer, gray contrast shows monolayer graphene, and white patches indicate bilayer graphene. The polymer amount on the SiC surface was varied by different isopropanol rinsing times as denoted in the columns. (a) and (c) Show results for the longer rinsing time of 10–12 s. Longer rinsing leads to lower polymer content, leading to higher percentages of buffer and bilayer. On the high-miscut substrate (c), step bunching has taken place which favors the growth of elongated bilayer stripes along the terrace steps. (b) and (d) Show results obtained by optimum rinsing times of 6–8 s. A homogenous graphene monolayer (gray) is observed in both (b) and (d). Buffer layer and bilayer spots as observed in (b) but disappear on the higher-miscut substrate due to an enhanced carbon supply from step edge decomposition.

First, the graphene growth results for the polymer deposition by LPD technique are presented. Since the IPA rinsing after the polymer application is the most critical step, the rinsing time was varied and investigated for two 6H-SiC wafers with low (−0.03°) and high (−0.1°) miscuts. The surface morphologies of the final graphene layers were visualized by confocal laser scanning microscopy images (CLSM) (see Figure 1).

There are three major contrasts that can be observed in Figure 1a–d. The desired monolayer graphene, which has a smooth surface morphology, is revealed by the gray contrast. Note that for epitaxial graphene growth, the so-called zeroth graphene or buffer layer exists below the van der Waals bonded graphene layer. This buffer layer is covalently bonded to the substrate and is electronically inactive. The dark gray or blackish contrast exhibits the buffer layer that is not covered with graphene. In these CLSM images, the white contrast denotes bilayer graphene (overgrown) that is often formed at high step edges and can give rise to poor transport properties.^19,28^Figure 1 shows that for longer rinsing times (10–12 s; see Figure 1a,c), a much stronger contrast is observed compared to the shorter rinsing time (6–8 s; see Figure 1b,d). The stronger contrast is related to graphene bilayer and buffer layer coverage for both types of low and high-miscut wafers. Obviously, a longer rinsing time is not optimal for the graphene formation. This is in good agreement with the PASG model, since after a longer rinsing time, less polymer remains on the SiC surface, which results in a lower extra carbon supply during buffer layer and graphene growth. Therefore, step bunching is enhanced, which favors bilayer growth and thus leads to the formation of a discontinuous/nonuniform graphene monolayer. The elongated bilayer stripes (white contrast) observed in Figure 1c are typical for substrates with a higher miscut, on which step flow growth results in high terrace step edges decorated with bilayer stripes along the edges.

The difference between longer and shorter (optimal) rinsing using isopropanol shows the impact of the polymer amount on the quality using two different miscut substrates. For the shorter (optimal) rinsing time (6–8 s), shown in Figure 1b,d, a uniform monolayer graphene (gray contrast) coverage is observed. Here, the remaining extra amount of polymer on the surface results in a higher carbon supply during graphenization. This leads to a faster buffer layer growth, stabilization of the SiC terraces, and suppression of step bunching. The higher carbon amount also favors a homogeneous growth of the graphene layer after the buffer layer has formed. However, it should be highlighted that there is an observable difference between the low- and high-miscut-angle wafers, meaning the supply of available carbon from the decomposition of the underlying substrate itself is the other important parameter. Small, isolated buffer layer spots (dark contrast) remain after the formation of the graphene layer, and a few scattered bilayer patches (light gray contrast) are observed on the low-miscut substrate in Figure 1b. Since the decomposition of the SiC substrate is favored at terrace step edges and its amount increases with increasing miscut angle, a higher substrate-related carbon supply is present in the case of a high-miscut wafer. Therefore, the graphene on the higher-miscut substrate (Figure 1d) shows fewer buffer layer spots and no bilayer patches compared to Figure 1b.

This comparison demonstrates that the polymer amount that determines how much carbon is supplied to obtain an optimal growth result must be adapted to the substrate’s miscut angle. However, the main control of the amount of polymer adsorbates is decided by the rinsing step, which is manually operated and thus user dependent and challenging to reproduce. Since the sample handling during rinsing is critical to the growth result, the LPD technique is limited when it comes to the growth optimization on SiC substrates with different properties and when it comes to the operation of different users. Apart from the CLSM images, SEM, AFM, and Raman data of these samples are provided in Figures S1–S4 in the Supporting Information for a better understanding of the critical steps in the LPD technique.

**Figure 2:**
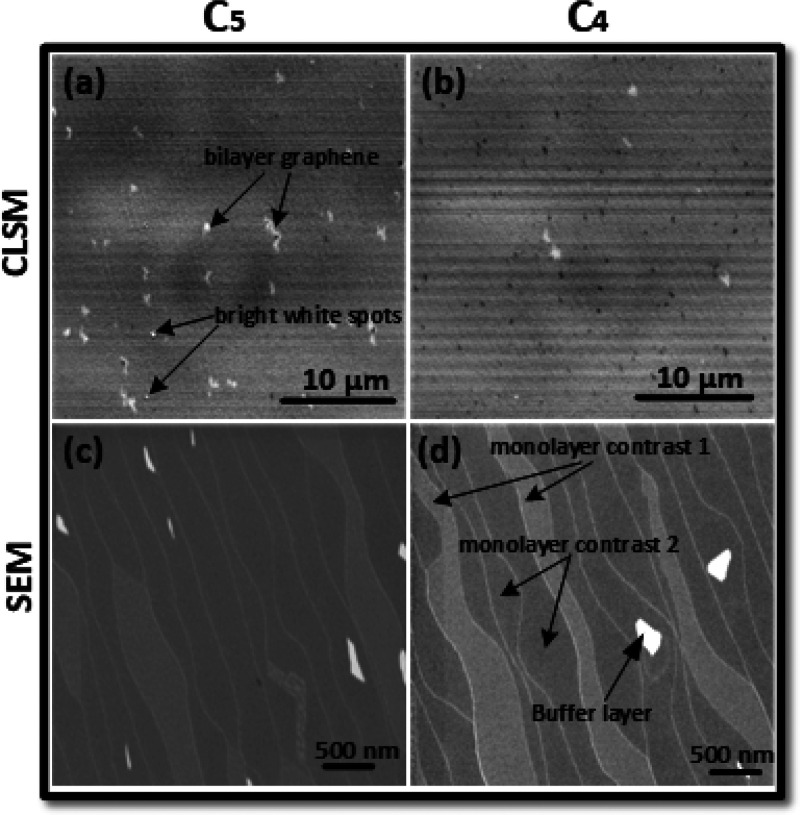
Graphene samples processed by spin-on deposition of the polymer with high concentrations C5 and C4 on 6H-SiC with a miscut of −0.1°. (a, b) CLSM images of epitaxial graphene using concentrations C5 and C4. The isolated white patches indicate graphene bilayers. The bright white spots are ascribed to polymer aggregates. (c, d) SEM images of the same samples with a higher resolution. The SEM contrast is opposite to that of CLSM: i.e., the white patches in SEM indicate the buffer layer. The contrast between monolayer graphene on adjacent terraces is related to differences in the polarization doping of the underlying SiC terraces.

**Figure 3:**
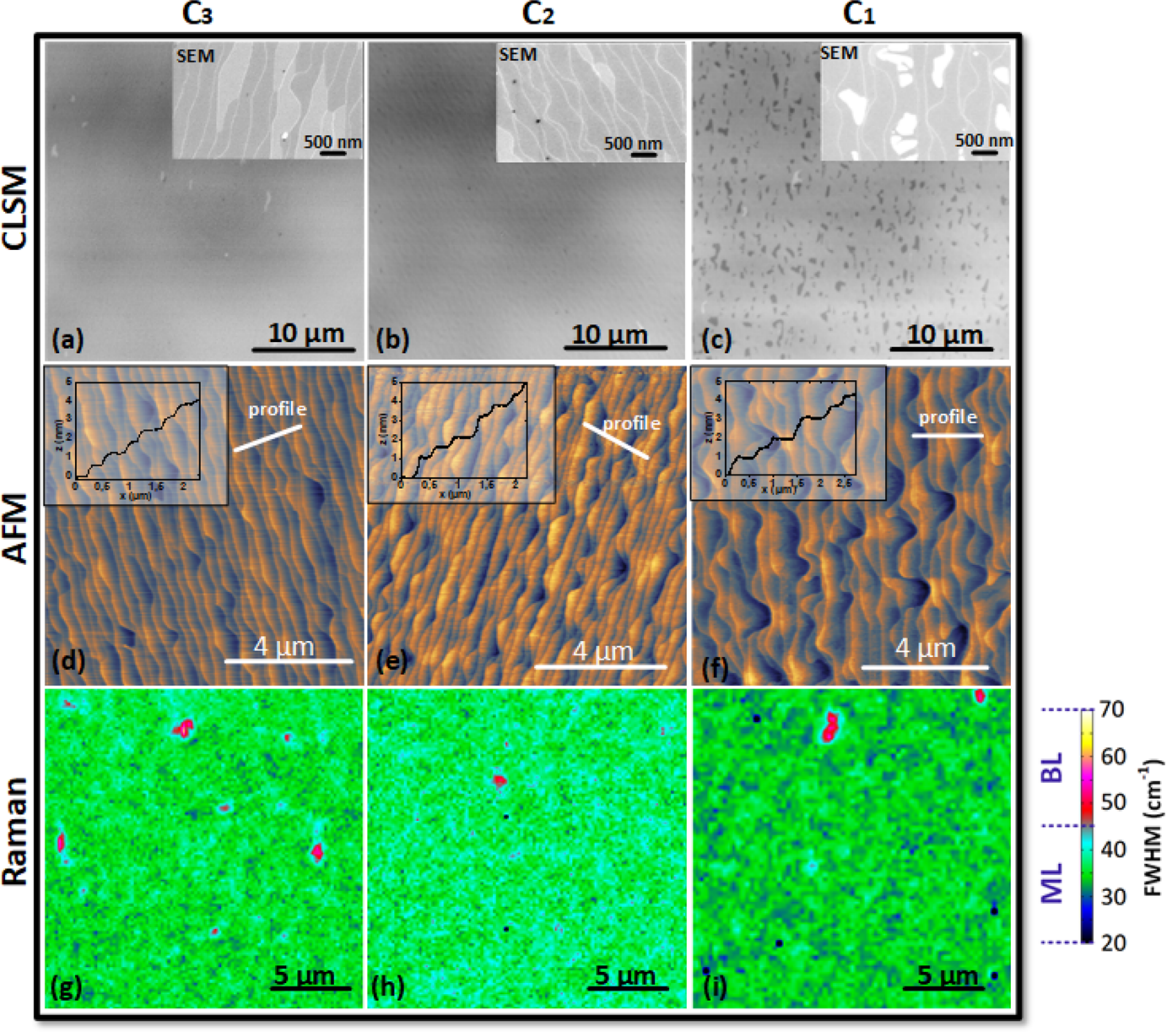
Graphene samples processed by spin-on deposition of the polymer with concentrations C3–C1 using 6H-SiC substrates with a miscut of −0.1°. (a–c) CLSM images of epitaxial graphene using polymer concentrations C3–C1, respectively. The insets show SEM images of the same samples. Note that buffer layer areas in (c) have a dark contrast in CLSM and a white contrast in SEM. (d–f) AFM topography images of the same samples. The color height scale is 0–2.5 for all images. Insets show the height profile along the marked white line. (g–i) Micro-Raman maps (20 μm × 20 μm) of the 2D peak width (fwhm) extracted from 7000 individual Raman spectra of each graphene sample. The surface is covered with very homogeneous monolayer graphene, which is indicated by the narrow fwhm of 30–40 cm^–1^ (blue and green areas). The small isolated red spots (fwhm ∼50 cm^–1^) originate from bilayer graphene.

**Figure 4:**
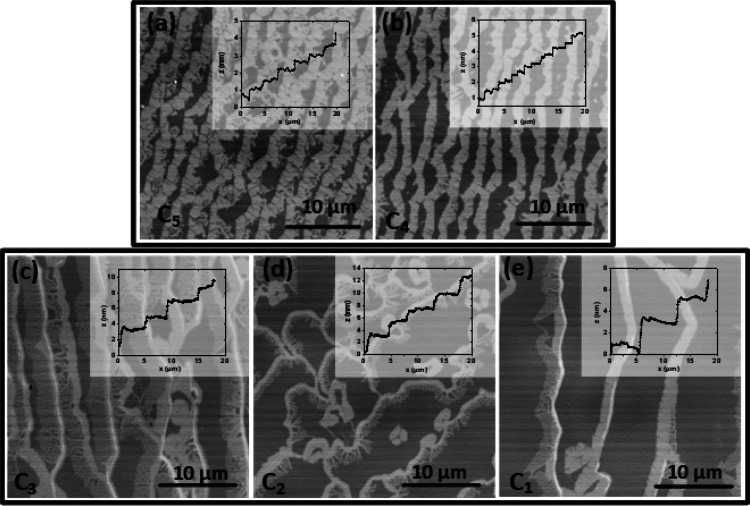
Graphene samples processed by spin-on deposition of polymer on 6H-SiC substrates with a small miscut of −0.03°. (a–e) Variation of the polymer concentration from high C5 to low C1, respectively. The graphene coverage decreases toward lower polymer concentrations, indicating a lower carbon supply. At higher concentrations (a, b), a higher carbon supply from the polymer induces terrace nucleation and prevents step bunching. At lower polymer concentrations (c–e) the wider terraces indicate step bunching and fingerlike graphene structures developing perpendicularly to the terraces.

**Figure 5:**
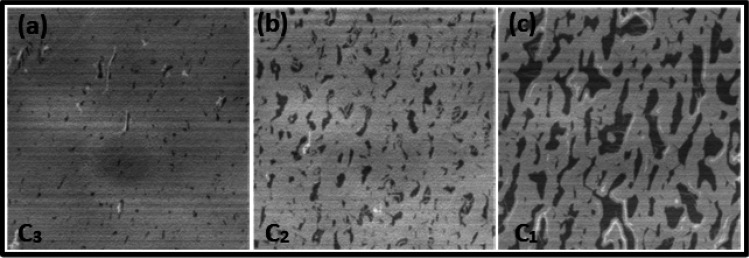
CLSM images of graphene samples processed by spin-on deposition with different polymer concentrations C3–C1 on a 4H-SiC substrate with a miscut angle of +0.04° toward the primary flat. (a) At the moderate polymer concentration C3 a nearly homogeneous graphene monolayer (gray) has formed. A few small islands with a buffer layer (dark spots) remain uncovered. (b) With decreased polymer concentration C2, the area of the buffer layer patches (dark gray) increases. (c) At the lowest polymer concentration, C1, large buffer layer patches remain uncovered. The CLSM images show that the homogeneity of monolayer graphene decreases with decreasing polymer concentration, indicating the reduced external carbon supply for graphene growth.

In the following, we show the results of PASG of epitaxial graphene with spin-on deposition of the polymer on the SiC substrate, which allows for improved controllability and reproducibility. First, the impact of different polymer amounts on the graphene morphology is exemplarily examined by using a 6H-SiC wafer with a −0.1° miscut (see Figures 2 and 3). Then, the results of spin-on deposition on a lower miscut 6H-SiC substrate and a 4H-SiC polytype are presented and discussed (see Figures 4 and 5).

Figure 2 shows the CLSM and SEM images of epitaxial graphene on 6H-SiC with a miscut angle of −0.1° toward the primary flat for the highest polymer concentrations, C5 and C4. The CLSM images in Figure 2a,b show a homogeneous monolayer graphene coverage of 99% of the area for both polymer concentrations, which has been confirmed by Raman measurements. However, for the highest concentration (Figure 2a), a high density of bilayer patches (white) is observed. For the lower polymer concentration (Figure 2b), the bilayer density is reduced but dark spots identify areas of uncovered buffer layer. Moreover, especially for the highest polymer concentration (Figure 2a), some very bright white spots are observed that are the signature of polymer aggregates. This indicates that there is a surplus of polymer-related carbon and that the maximum polymer concentration for this miscut angle is reached. In view of the small difference of the polymer concentrations between C5 and C4, the observed differences in the graphene morphology underline the reproducibility of the spin-on deposition technique, which allows a good control of the quality of the final graphene layer.

Figure 2c,d shows the SEM images of the same samples with much higher spatial resolution. At the low SEM acceleration voltages used, long stripes of binary gray contrast with a width of several 100 nm can be distinguished for the large areas of monolayer graphene (monolayer contrasts 1 and 2). This interesting feature is caused by the different surface polarizations of the underlying SiC terrace terminations.^29^ The contrast identifies graphene on terraces with different layer terminations within the 6H-SiC unit cell. It is also an indication of very low step heights of one or two SiC layers (0.25 and 0.5 nm), and it proves that step bunching was effectively suppressed. The bright line contrast reveals terrace step edges between SiC terraces with equivalent terminations with a height of a half 6H-SiC unit cell, ∼0.75 nm.^29^ Note that in the SEM images the buffer layer appears white (white spots) and bilayer graphene is hard to distinguish from the monolayer because of a small work function difference.^27^ Although the investigation of the high polymer concentrations C5 and C4 for PASG has shown that very smooth monolayer graphene can be obtained, the formation of a buffer layer and polymer aggregates indicates that these are not the optimum concentrations for this wafer.

In the following, the growth results for the remaining three polymer concentrations C3–C1 for the same wafer are analyzed (see Figure 3).

The CLSM images for the moderate polymer concentrations C3 and C2 shown in Figure 3a,b reveal a homogeneous coverage of monolayer graphene. For C3 only a few bilayer patches exist, which completely disappear at the lower concentration C2. For the further reduced polymer concentration C1 (volume ratio of 0.75; Figure 3c), a significant density of large buffer layer patches (dark gray) can be noticed in the CLSM image and in the SEM enlargement (white), as displayed in the inset. Already from the CLSM inspection we can conclude that homogeneous large-area monolayer graphene is obtained by the moderate polymer concentrations C3 and C2 for this wafer miscut.

A comparison of the surface morphology from the SEM images for the polymer concentration series (see Figure 2c,d and insets of Figure 3(a–c) shows another interesting feature: namely, that the curviness of the terrace edges increases with reduced polymer concentration. This evolution of the terrace edges is also clearly visible in the AFM images for the polymer concentrations C3–C1 shown in Figure 3(d–f). This is attributed to the difference in the amount of polymer on the surface (as adsorbates) that changes the decomposition rates of the active carbon species. Previous studies have shown (even without polymer) that the growth involves numerous competing processes leading to different speeds of carbon diffusion on the surface.^30^ With the additional carbon that is externally applied through the polymer in our study, we can conclude that an optimal concentration of carbon supports a uniform decomposition of the SiC surface, well-defined terrace steps, and an improved parallelity of the terraces. Therefore, moving away from the optimum concentration C3 makes the terraces curvier in nature.

The AFM images in Figure 3d–f shows the evolution of the graphene topography when the polymer concentration is reduced. All AFM scans were performed on 10 μm × 10 μm areas, and the height scale ranged from 0 to 2.5 nm. The insets show the profile of terrace height and widths extracted from the AFM scans along the marked line.

Figure 3d shows that concentration C3 results in very regular step heights of mainly 0.25 and 0.5 nm (corresponding to one and two SiC layers). The low step heights are also reflected in the binary SEM contrast of the monolayer graphene induced by different surface polarization of the underlying nonequivalent SiC stack terminations of terraces with a height difference of one or two SiC layers. Over the complete area, an alternating pattern of terrace widths of about 200 and 300 nm is observed. For the lower polymer concentrations C2 and C1 (Figure 3e,f), the step height distribution increases and also higher steps of 1 nm are observed. Accordingly, wider terraces are observed, and their widths vary between 200 and 400 nm. This comparison shows that the polymer concentration can also be used as a parameter to fine-tune the terrace properties: e.g., the widths, heights, and parallelity of the terraces.

In Figure 3 (g–i), micro-Raman mappings of an area of 20 μm × 20 μm are displayed. They show color plots of the full width at half-maximum (FWHM) value of the so-called 2D peak of graphene, from which the number of graphene layers and the homogeneity can be identified.^31,32^ Examples of representative Raman spectra for these three samples are shown in Figure S5 in the Supporting Information. From the color scale, it can be observed that the 2D-peak FWHM values for our samples C3 and C2 lie in the range of 30–40 cm^–1^ (green color). From the Gaussian frequency distribution of the FWHM values a mean value of 34 ± 2 cm^–1^ was extracted, which proves the high quality of the graphene monolayers.^10^ The incomplete graphene layer of the sample with the lowest polymer concentration, C1, shows areas with a narrow fwhm of ∼30 cm^–1^ (black and dark blue patches in Figure 3i), which are attributed to the buffer layer.

As we use our graphene for metrological purposes and for fabricating devices to perform precision quantum Hall measurements for resistance and impedance standards,^24,33,34^ Raman is an essential prerequisite for the quality control of our graphene samples.^24,26^ In the Raman mappings also small, isolated spots with a broadened 2D FWHM of about 50 cm^–1^ (red) are observed. They are attributed to small graphene bilayer domains, in agreement with the CLSM and the SEM images, and they have no detrimental effect on the resistance standards.^33^ For the best samples (for example C3 and C2) mobilities are in the range ∼5000–7000 cm^2^/(V s) and the typical charge carrier densities are close to ∼5 × 10^11^ cm^–2^ after lithography and no additional doping of the processed graphene devices. These are essential criteria for the fabrication of electrical devices such as electrical resistance standards for our calibration needs in metrology. The values suggest that high-quality homogeneous graphene monolayer films have been made that are suitable for these magnetotransport precision measurement applications.^33,34^

The examination of the monolayer coverage for the overall polymer concentration series from high to low values, C5 to C1, gives a consistent picture from slightly overgrown to undergrown. It is in very good agreement with the model that the polymer acts as an additional carbon source for graphene growth and that the graphene quality can be controlled by fine-tuning the polymer concentration. After a detailed analysis of the graphene growth on substrates with a miscut angle of 0.1° toward primary, it is interesting to investigate the influence of miscut variations also for the polymer spin-on technique.

To verify our hypothesis of a correlation between miscut angle and polymer concentration derived from the previous results for 0.1° miscut angles shown above, we also extended our spin-on deposition technique to 6H-SiC substrates with miscuts higher and lower than 0.1°. For the higher miscut angle of −0.3° toward primary flat, concentration C2 was sufficient to get fully covered high-quality graphene monolayers, whereas for the 0.1° miscut, the polymer concentration C3 was optimum. This shows already that a higher miscut angle requires a lower absolute polymer concentration to produce equally good graphene monolayers. The results for the high-miscut −0.3° 6H-SiC with C2 concentration are provided in Figure S6 in of the Supporting Information. This trend is in very good agreement with the model that higher miscut substrates with higher step density can supply more carbon by thermal decomposition of SiC at the terrace edges and therefore require less polymer externally. For miscut values lower than 0.1°, the results are described below in detail.

In the following, we concentrate on the growth results on 6H-SiC substrates with a very low miscut angle of −0.03°. Figure 4(a–e) shows the CLSM images of the graphene samples with decreasing polymer concentrations from C5 to C1. For the lowest polymer concentration, C1 (see Figure 4e), narrow graphene domains (light gray stripes) along the step edges are observed, indicating a very low carbon supply that is related not only to the low polymer concentration but also to the small miscut angle, since from the corresponding fewer steps (compared to higher-miscut substrates) less carbon is released by SiC step edge decomposition. The dark gray contrast on the terraces reveals that most of the area is covered only with a buffer layer. The broad, nearly 5 μm wide terraces as well as step heights of several nanometers measured by AFM reveal that step bunching has taken place during graphene growth for the lowest concentration. The white stripes indicate the step-bunching effect, and insets show the height profiles obtained from AFM scans.

With an increase in polymer concentration to C2 and C3 (see Figure 4c,d), the terrace width starts to decrease, and the AFM height profiles reveal lower step heights: i.e., step bunching is reduced which is caused by the higher polymer-related carbon supply that stabilizes the SiC surface by accelerated buffer layer growth. Figure 4d shows an interesting intermediate step of graphene island growth on the terraces, which is attributed to an increased number of polymer-related nucleation centers. The incomplete graphene growth reveals fingerlike structures of nanometer-size graphene nanoribbons/graphene fingers (see Figure 4c) of 200–300 nm (measured from AFM phase images) which have grown perpendicularly to the terrace edges.^16^

At the higher polymer concentrations C4 and C5 (Figure 4a,b), continuous and wider graphene domains (light gray stripes) have formed along the terrace edges and a higher surface density of terraces is seen. The overall trend is that the area covered with graphene is increasing with the higher polymer concentrations, which is attributed to the enhanced polymer-related carbon supply. The extra terraces appear due to carbon nucleation from the polymer (so-called “terrace nucleation”^15^). However, even for the highest polymer concentration, areas with a buffer layer (dark gray) remain uncovered with graphene. This is in contrast to the complete graphene coverage on substrates with higher miscut angle of 0.1° (see Figure 3), when the same polymer concentrations are used. This clearly indicates an insufficient carbon supply from SiC step edge decomposition for a low step density on a substrate with a small miscut angle. The observation confirms the assumption that, next to the polymer concentration, also the SiC decomposition at the step edges plays an important role in graphene growth. This means that, for optimal homogeneous graphene growth, the polymer concentration must be adapted to the miscut of the wafer. Note that, even though the graphene layer growth at high concentrations C5 and C4 is still incomplete, the graphene stripes at the step edges have successfully suppressed step bunching (very small step heights as confirmed from AFM profiles and the absence of white stripes in CLSM). The AFM height profiles in the insets reveal step heights of 0.25 and 0.5 nm, which correspond to one and two SiC layers, respectively, as shown in the inset of Figure 4b.

Another interesting feature is that even though the polymer concentration in Figure 4a is insufficient for complete monolayer coverage on this wafer as discussed above, Figure 4a (similarly to Figure 2a) shows bright white spots in CLSM and AFM indicating polymer aggregates on the surface. This means that the C5 concentration is also the highest for low-miscut wafers, and the remaining carbon needs to be supplied from the SiC wafer. From AFM profiles shown in the inset of Figure 4b, it can be identified that the C4 concentration is able to produce ultralow step heights and is the optimum concentration for this wafer with a miscut angle of 0.03°. In order to get complete graphene monolayers for such low-miscut wafers, the growth temperature and/or time have to be increased. From this study, it is clear that the interdependence/balance between the two types of carbon supply can control very well the step heights and graphene morphology during growth. After comparing different miscut angle wafers of 6H-SiC, the last comparison would be on polytypes.

The last series in Figure 5 compares the effect of different polymer concentrations on a 4H polytype SiC substrate. Figure 5(a–c) represents the CLSM images of the 4H wafer with a miscut angle of +0.04° toward the primary flat. As shown in Figure 5a, the C3 concentration produces quite homogeneous graphene monolayers, with an almost negligible amount of buffer and bilayer patches. As we move to lower concentrations as in Figure 5b,c, the monolayer coverage decreases again, explaining the impact of a reduced external carbon supply on the graphene growth.

This tendency is the same as that observed for the graphene growth on 6H-SiC substrates. However, there is a noticeable difference between the growth results on the two polytypes for the same polymer concentrations. For the same concentration C3 applied to the 6H-SiC substrate with a comparable miscut angle, an incomplete graphene layer has formed (see Figure 4c). This indicates a higher intrinsic carbon supply when using a 4H-SiC substrate that can be explained by different decomposition velocities of 4H- and 6H-SiC polytypes, as has also been suggested in the literature.^35^ Since we used the same growth conditions, our observations confirm that the decomposition of a positive miscut 4H wafer (+0.04°) is faster than that of a comparable value but negative miscut 6H polytype (−0.03°). The influence of the miscut direction is not analyzed here. The overall trend of the effect of polymer concentration on miscut angle remains unaltered for both polytypes.

## 4 Conclusions

In this work, we demonstrated the concentration-dependent effect of polymer-treated SiC substrates on the graphene growth quality using spin-on deposition for the PASG technique. The prerequisite for homogeneous and complete graphene layers involves a complex interplay of carbon supply from the substrate and the external source. The observations demonstrate that low polymer concentrations may lead to incomplete graphene monolayer coverage and that giant step bunching may occur, whereas when the concentration is optimal, the layer becomes more and more complete. On the other hand, an excess of externally applied carbon can lead to polymer aggregates and other growth defects. Such fine control on the layer quality is the main advantage of this spin-on deposition technique. For each SiC polytype, there are differences in the optimum amount of externally applied carbon to effectively suppress step bunching during the graphene growth process. The concentration of polymer that is necessary for an ideal monolayer coverage depends significantly on the miscut angle of the SiC wafer. The surface density of step edges defines the number of weak points in the crystal and in turn the miscut significantly affects the amount of carbon that is released during the decomposition of the SiC wafer surface. The findings also suggest that the required polymer concentration also depends on the polytype. 4H-SiC requires less carbon from the polymer in comparison to its 6H equivalent for a complete monolayer graphene coverage. This study of the dependence on graphene quality with respect to the substrate properties and external carbon supply by using PASG is an important baseline for optimizing the control of the graphene morphology for electronics, surface science, and intercalation applications.

## Supporting Information Available

Representative Raman spectra of samples processed by the LPD technique, Raman 2D maps and respective histograms, AFM topography of samples processed by the LPD technique, SEM of samples processed by the LPD technique, representative Raman spectra of samples processed by the spin-on deposition technique, and growth results of a high-miscut (−0.3°) 6H wafer (PDF)
