# Supplementary material for: Impact of Polymer-Assisted Epitaxial Graphene Growth on Various Types of SiC Substrates
Source: ACS Appl Electron Mater. 2022 Nov 1;4(11):5317–25. doi: 10.1021/acsaelm.2c00989 (PMC9686134; doi:10.1021/acsaelm.2c00989)
Supplement: Supplementary file 1 — el2c00989_si_001.pdf [file el2c00989_si_001.pdf]

Supporting Information

Impact of polymer assisted epitaxial graphene growth on various types of SiC substrates

Atasi Chatterjee; Mattias Kruskopf; Stefan Wundrack; Peter Hinze; Klaus Pierz; Rainer Stosch and Hansjoerg Scherer*

*Corresponding author email: atasi.chatterjee@ptb.de

Physikalisch-Technische Bundesanstalt, Bundesallee 100, 38116 Braunschweig, Germany

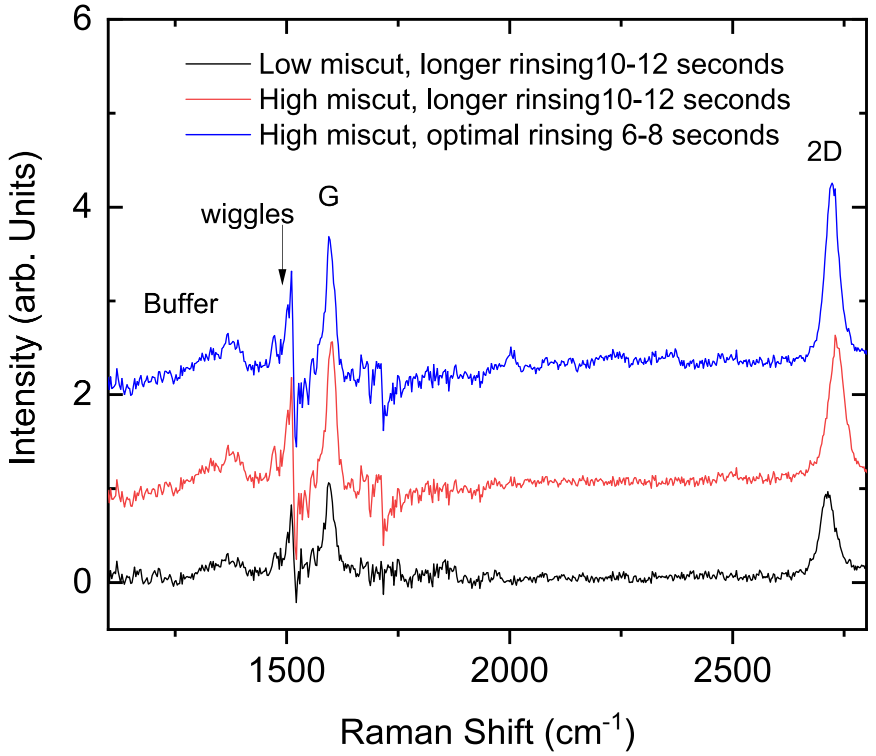

Figure S1: Raman spectra of graphene samples processed by LPD technique with longer (10-12 sec) and optimum rinsing times (6-8 sec) on low and high miscut 6H-SiC substrates.

Figure S1 shows the individual Raman spectra of the three out of four samples shown in Figure 1 of the main manuscript. The individual Raman spectra do not provide complete information on the total sample, but from the intensity difference of the 2D peak, it can be identified that the black curve which is the low miscut sample with a longer (10-12 sec) rinsing time has lower 2D peak intensity and is slightly shifted to lower wavenumbers. It is also broader in nature as this sample has a mixture of buffer, mono and bi-layer patches as can be seen from the CLSM images in Figure 1 of the main manuscript. The red and blue curves are for the high miscut wafer with longer and optimal rinsing times, respectively, showing that the best monolayer graphene coverage shows the highest intensity of the 2D peak.

The wiggles marked in the images arise from the subtraction artifacts. A clean and unprocessed 6H-SiC reference sample is used for background subtraction to reduce signal to noise ratio of the acquired Raman spectrum. This may introduce spectral artifacts @1500cm^{-1}, most likely occurring from the uncertainties in Raman spectral calibration.

Figure S2 shows the Raman mappings and the corresponding histograms of the samples prepared by LPD technique. Figure S2 clearly shows the difference between rinsing times and miscut angles, which is discussed in the main manuscript under Figure 1. Raman mappings provide information on the overall homogeneity of the monolayers, which is very important for our Quantum Hall measurements.

When the miscut angle of the wafer is low, additional carbon compensation is required by the polymer treatment, which is controlled by the rinsing step. Therefore, the overall remaining carbon after the polymer treatment process, decides the amount of graphene coverage.

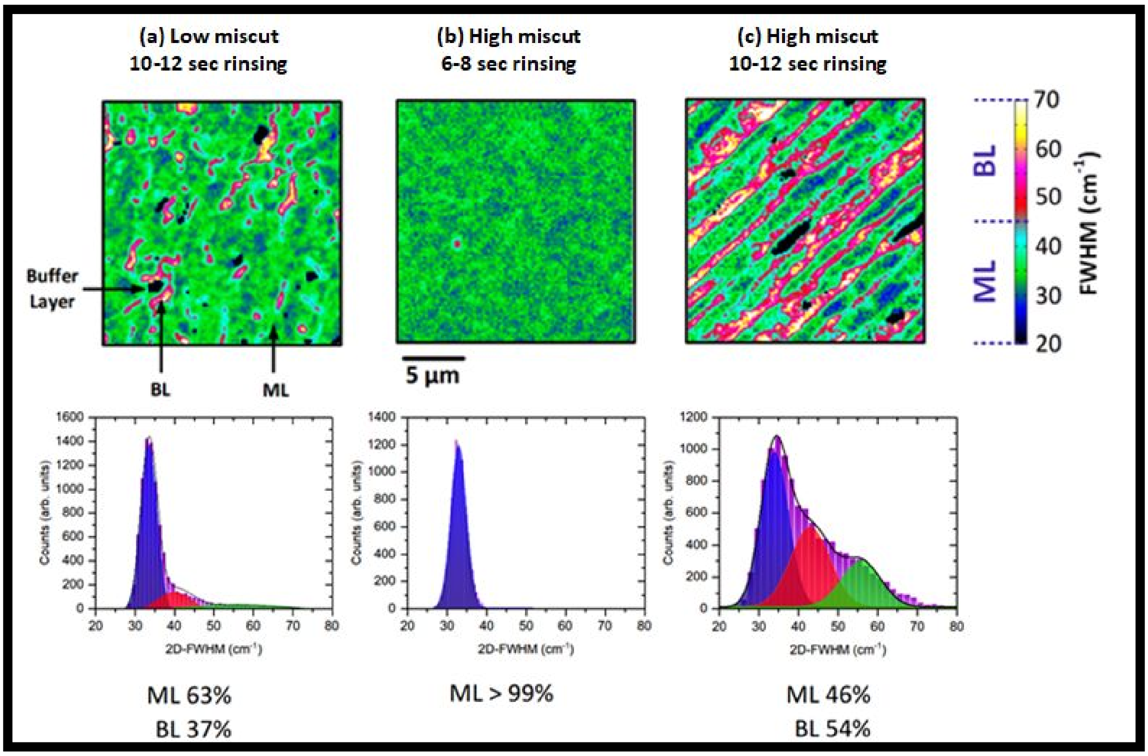

Figure S2: Raman mapping and respective histograms of graphene samples processed by LPD technique of (a) low miscut and longer (10-12 sec) rinsing (b) high miscut and optimum rinsing times (6-8 sec) and (c) high miscut and longer (10-12 sec) rinsing times on 6H-SiC substrates.

Only optimum amount of carbon adsorbates available together from the wafer and external source can lead ultra-low step heights and avoid step bunching to produce to uniform and homogeneous monolayer graphene. This condition is fulfilled in Figure S2 (b), where the amount of carbon from the wafer and optimum rinsing leads to the right amount of carbon needed to seed uniform nucleation. The provided results using the LPD technique are representative examples.

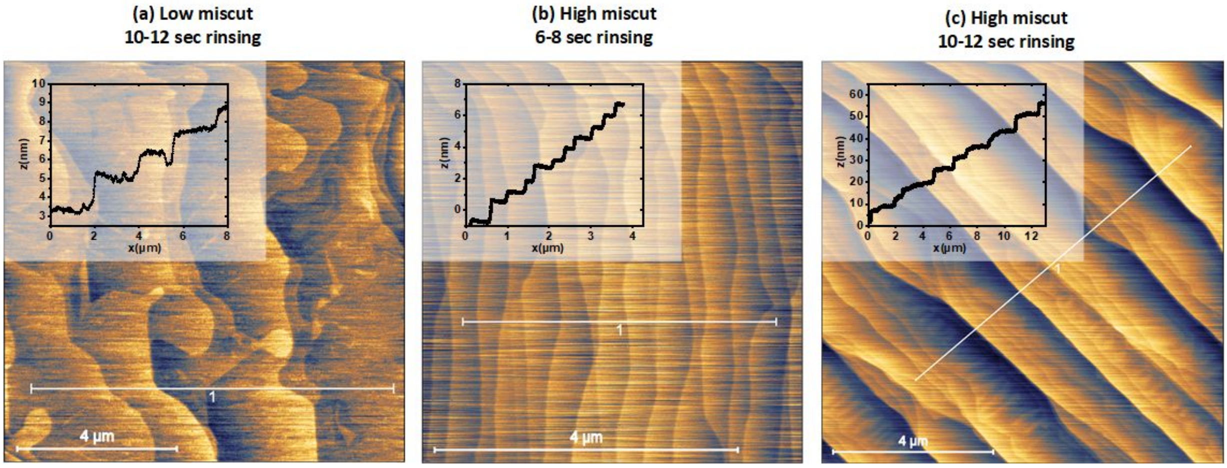

Figure S3: AFM topography of graphene samples processed by LPD technique with longer (10-12 sec) and optimum rinsing times (6-8 sec) on low and high miscut 6H-SiC substrates. (a) low miscut and longer (10-12 sec) rinsing (b) high miscut and optimum rinsing times (6-8 sec) and (c) high miscut and longer (10-12 sec) rinsing times. The AFM images depict the topography and the corresponding line profiles are shown in the insets of the AFM images.

AFM topography of the above samples show clearly the importance of the rinsing step and the effect of miscut angles. In Figure S3 (a) the low miscut wafer and longer rinsing times (10-12 sec) lead to insufficient carbon supply from both sources, resulting into broad terraces (approx. 1.5-2µm) and high step bunching upto several nanometers, leading to irregular graphene growth. Similarly, for a high miscut wafer e.g. in (c), excess supply of carbon from the wafer causes extreme high step bunching (~10 nm high steps) and formation of bilayers along the step edges, but longer rinsing times results in insufficient carbon within the steps providing almost no nucleation within the terraces for initial growth stages of the buffer layer. In Figure S3 (b) it is clear that the balance of carbon supply from the wafer and carbon from the polymer is in optimum quantity, giving rise to very small step heights of 0.5 and 0.75nm and 200-300nm broad terraces.

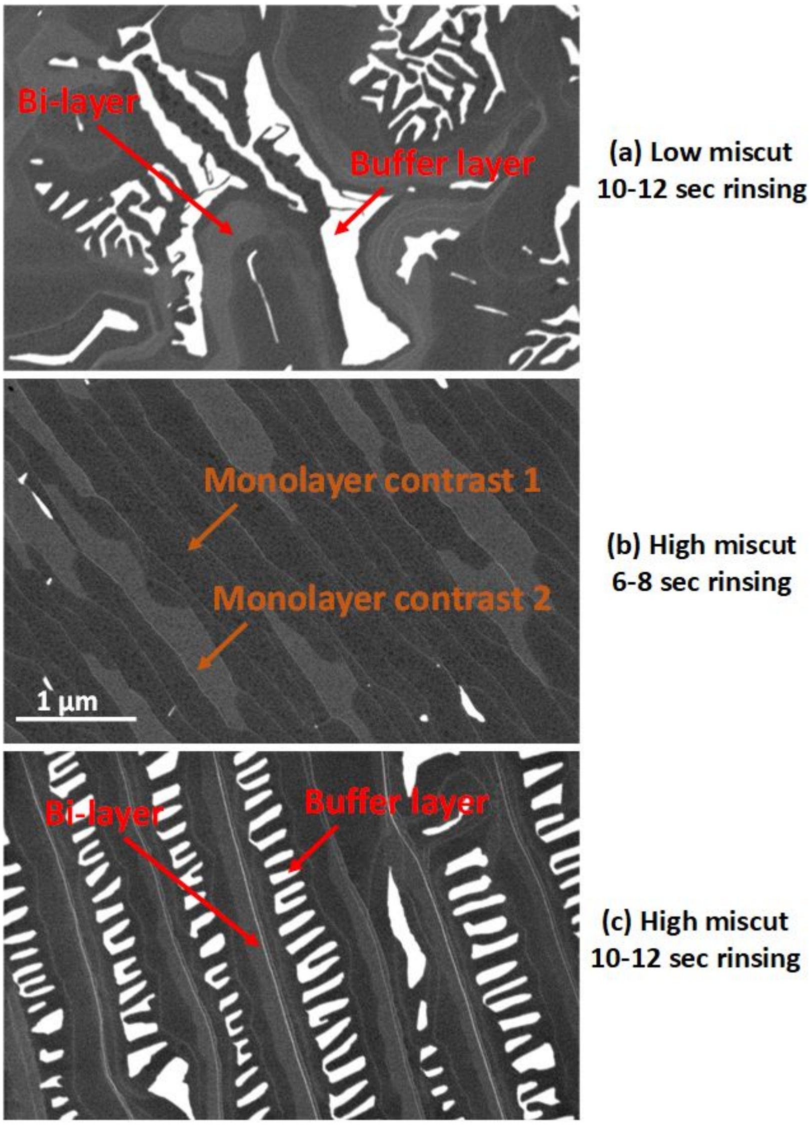

Figure S4: SEM of graphene samples processed by LPD technique with longer (10-12 sec) and optimum rinsing times (6-8 sec) on low and high miscut 6H-SiC substrates. (a) low miscut and longer (10-12 sec) rinsing (b) high miscut and optimum rinsing times (6-8 sec) and (c) high miscut and longer (10-12 sec) rinsing times.

Figure S4 shows SEM images at low electron energies for a zoomed in look at the morphological structures. This shows in detail, how the surface is modified with different contrasts for different graphene layers corresponding to the LPD technique. The white

contrast in Figure S4 (a) and (c) marks the buffer layer covered areas and two different grey contrast in Figure S4 (b) shows two different monolayers. Scattered white patches in (b) are also buffer layers. Interesting to see that the bilayer contrast in SEM is very close to monolayer graphene contrast 2, due to very close values of work functions. Apart from the explanation of figures, overall we see very good quality monolayer graphene in Figure S4 (b) due to optimum carbon supply from the combined effect of miscut and polymer concentrations as explained in detail in the main manuscript.

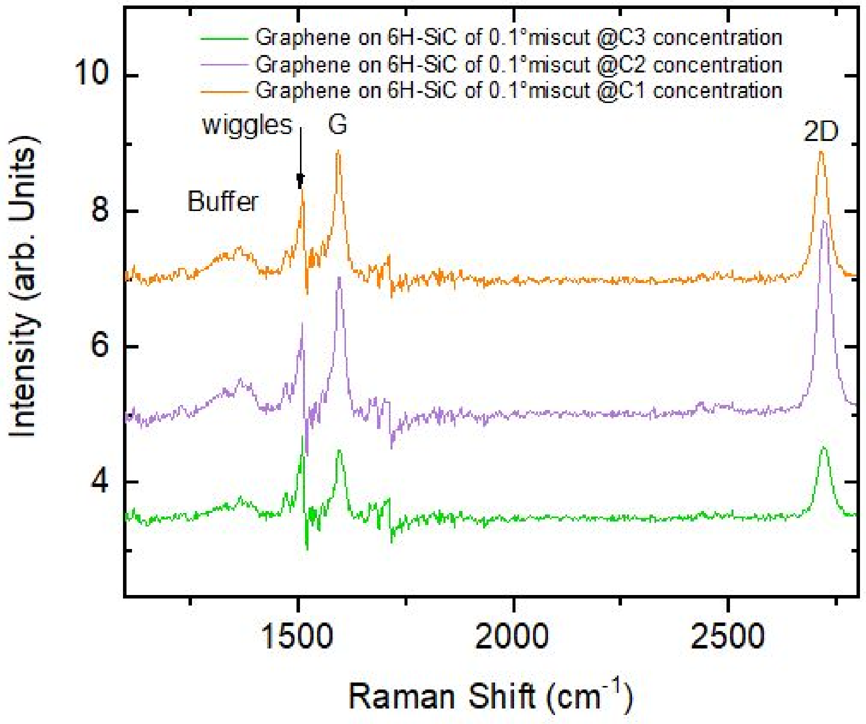

<u>**Figure S5**: Raman spectra of graphene samples processed by spin-on deposition technique of 6H-SiC substrates (-0.1° miscut towards primary) with C3 , C2 and C1 concentrations</u>

From Figures S1 and S5 it can be concluded that the intensity of the Raman signal is much higher in case of spin-on deposition samples in comparison to the LPD technique. Also, all the three samples show very good 2D peak structures of monolayer graphene. The intensity of 2D peak of graphene for C2 concentration is the highest followed by C3 and C1. The intensity of 2D peak for C3 and C2 is almost comparable. This also suggest that for C3 and C2, graphene quality is perfect. Such homogeneous monolayer graphene quality is very important for our metrological applications. As we use our graphene to prepare Hall bar devices for resistance and impedance standards, therefore, the overall homogeneity of the samples are quite important for us. For this reason, Raman mappings are very important in this case. It is more representative regarding the coverage over the whole sample, as shown in Figure 3 of the main manuscript. The wiggles are observed due the same reason as explained in detail in the explanation of Figure S1.

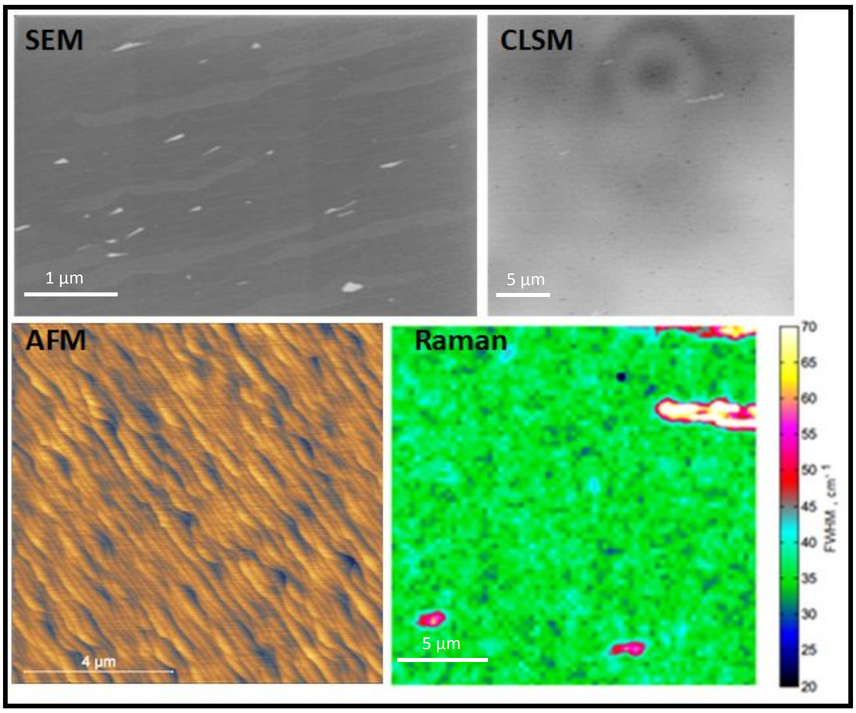

Figure S6: CLSM, SEM, Raman data and AFM topography of graphene grown on 6H-SiC with very high miscut towards the primary (-0.3°) using spin-on deposition @concentration C2.

Figure S6 shows an example of monolayer graphene grown on 6H-SiC with a very high miscut wafer (0.3°towards primary flat) but using the concentration C2. The miscut was confirmed from AFM investigations. From SEM and AFM, narrow terraces are observed as a characteristic of high miscut samples. From SEM, again two different terrace contrasts can be identified for monolayer graphene with different underlying surface terminations.

For miscut of 0.1°towards primary flat, C3 concentration was enough but this result shows that, higher the miscut, the lower is the requirement of carbon from the external polymer. Therefore, for this sample (-0.3°), even lower concentration C2 was enough to produce

equally good graphene. But for even low miscut wafers, even concentrations C4 and C5 were needed as shown in detail in the manuscript.
